# Analysis of Bone Loss around Cemented and Biologic Prostheses after Hemiarthroplasty

**DOI:** 10.1155/2022/7366576

**Published:** 2022-08-05

**Authors:** Fan Zhang, Chao Liu, Haibo Xu, Xiafen Zhang, Hanliang He

**Affiliations:** ^1^The Department of Orthopedic Surgery, Dushu Lake Hospital Affiliated to Soochow University, Suzhou, 215028 Jiangsu, China; ^2^The Department of Orthopedic Surgery, Suzhou BenQ Medical Center, The Affiliated BenQ Hospital of Nanjing Medical University, Suzhou, 215000 Jiangsu, China

## Abstract

**Objective:**

To perform hemiarthroplasty (HA) on elderly patients with femoral neck fractures using cemented and biologic prostheses and then compare the bone loss around the two types of prostheses after surgery.

**Methods:**

A total of 60 patients aged over 75 years (with a mean age of 83.5 years) and suffering from femoral neck fracture (Garden types III and IV) from January 2018 to December 2020 were selected; they were randomly divided into group A (*n* = 30, cemented prostheses) and group B (*n* = 30, biologic prostheses) and received HA. At 1 month, 6 months, and 12 months after surgery, Harris Hip Scale (HHS) was adopted for patient evaluation, and patients' bone mineral density (BMD) of the 7 Gruen zones around the prostheses was measured by dual-energy X-ray absorptiometry (DEXA scan).

**Results:**

Both groups achieved satisfactory results postoperatively, and the Harris scores of the hips increased gradually over time, which were better in group A than in group B. Gruen zones in group A showed a slower trend of decreasing BMD than group B, and the differences were significant at zones 2, 3, and 4 (*P* < 0.05).

**Conclusion:**

For elderly patients with femoral neck fractures, selecting cemented prosthesis for HA better recovers the hip function and has a low rate of bone loss around the prosthesis.

## 1. Introduction

Osteoporosis is increasingly recognized as the population ages. Patients with hip fracture caused by osteoporosis need long-term bed rest and have high rates of complications and mortality, resulting in great pressure on medical treatment and society. Currently, among the elderly hip fracture patients in China, the most common is femoral neck fracture, which accounts for 3.6% of all fractures and 45%-54% of hip fractures [1]. Osteoporosis prevention and treatment in elderly patients as well as performing bone mineral density (BMD) screening in a scientific and effective way has become the secondary prevention focus of orthopedics in China in recent years [2]. Currently, nondisplaced femoral neck fractures (Garden types I and II) can achieve satisfactory clinical results through internal fixation [3]; and although optimal treatment of displaced femoral neck fractures (Garden types III and IV) remains controversial, most choose hemiarthroplasty (HA) or total hip replacement. HA is technically simple; has short operation time, less blood loss, and low dislocation rate; and is suitable for elderly patients with displaced femoral neck fractures who have poor physical condition and less activity [4]. The cemented and biologic prostheses are two commonly used types, and the former case, as a mosaic agent, can firmly integrate the metal part of artificial joint with the patients' own bones, with the applied advantages of rapid postoperative recovery and small trauma, but the surgical procedures may damage the nerves, while the latter case with a short surgery time can reduce postoperative complications to a certain extent, but the probability of periprosthetic fracture is higher. Therefore, there is no consensus view on the choice of cemented or biologic prostheses. In this study, by adopting both cemented and biologic prostheses for HA in elderly patients with femoral neck fractures and recording postoperative improvement in hip function and changes in periprosthetic bone density, the clinical outcomes were evaluated.

## 2. Materials and Methods

### 2.1. Inclusion and Exclusion Criteria

Inclusion criteria were as follows: (1) the patients were over 75 years old and had femoral neck fractures (Garden types III and IV); and (2) before hospitalization, the patients could walk and carry out activities normally. Exclusion criteria were as follows: (1) the patients were complicated with a history of severe coxitis or rheumatoid arthritis; (2) the patients were complicated with a history of fractures at other sites; and (3) the patients had bone metabolic diseases.

### 2.2. General Data

A total of 60 patients with femoral neck fracture treated in our hospital from January 2018 to December 2020 were selected and randomly divided into group A (*n* = 30) and group B (*n* = 30); i.e., the samples were selected by the random number table. Experienced joint surgeons performed HA to patients in the two groups with the anesthesia method as tracheal intubation general anesthesia when patients were in lateral position by adopting the rear lateral approach and implanted the cemented and biologic prostheses according to the standard method [5]. After surgery, antibiotics were administered according to the clinical diagnosis and treatment code to prevent infection, deep vein thrombosis was prevented, and rehabilitation functional exercise was conducted. The CoCrMo alloy cemented femoral stem prostheses (morphologically matched type; specification: 127-933; NMPA approval No. 20173466557) were used in group A, and titanium alloy porous-coated cementless stem prostheses (tapered stem; specification: 1-4/129; NMPA approval No. 20203130412) were used in group B. The prostheses were provided by Zimmer (Shanghai) Medical International Trading Co., Ltd., and all patients were reviewed and approved by the Ethics Committee of BenQ Medical Center in Suzhou.

### 2.3. Evaluation Methods

The clinical data such as body mass index (BMI), surgery time, and hospitalization time were recorded, and the incidence of postoperative complications was counted. At 1 month, 6 months, and 12 months after surgery, follow-up visit was carried out, patients' Harris Hip Scale (HHS) scores were recorded, including four aspects of function, pain, malformation, and joint motion with a full score of 100 points in this scale and the score above 90 as excellent, 80-90 as better, 70-79 as acceptable, and less than 70 as poor, and patients' BMD of the 7 Gruen zones around the prostheses was measured by dual-energy X-ray absorptiometry (DEXA scan) for 3 times.

### 2.4. Statistical Analysis

Software SPSS 17.0 was used for data analysis, continuous variables were expressed as mean ± standard deviation, quantitative data that conformed to normal distribution were assessed by paired sample *t*-test, and those that have skewed distribution were assessed by Mann–Whitney *U* test at the test level *α* = 0.05, and differences were considered statistically significant at *P* < 0.05.

## 3. Results

### 3.1. Comparison of General Data

The patients' BMI and age were not statistically different between the two groups (see Table 1). The mean surgery time of group A was 70.13 ± 3.50 min, which was obviously higher than that of group B (52.52 ± 3.60 min, *P* < 0.001). No statistically significant difference on the mean hospitalization time between the two groups was observed (*P* = 0.443). A total of 5 patients had postoperative complications, 2 of them were in group A (1 with postoperative lower-extremity deep vein thrombosis and 1 with lung infection), and 3 of them were in group B (1 with postoperative lower-extremity deep vein thrombosis and 2 with urinary tract infection). All complications were cured after active treatment.

### 3.2. Postoperative Evaluation of Hip Joint Function

At 1 month, 6 months, and 12 months after surgery, the Harris scores of the hips increased gradually over time in the two groups. At postoperative 1 month, the Harris scores of patients in group A and group B were, respectively, 65.74 ± 5.32 points and 63.43 ± 5.76 points, presenting no statistical difference (*P* = 0.112), and at postoperative 6 months and 12 months, the Harris scores were better in group A than in group B. At postoperative 12 months, the Harris scores of group A and group B were, respectively, 84.56 ± 6.70 points and 80.29 ± 7.08 points, which reached a satisfied level (see Table 2).

### 3.3. Comparison of DEXA Analysis Results

Periprosthetic BMD loss was measured on 3 consecutive DEXA scans in all 7 Gruen zones, with group B specifically showing the highest degree of BMD loss in zone 7. The change in mean periprosthetic BMD values was different between groups A and B, with a slower trend towards lower BMD values in the Gruen zones in group A compared to group B using biologic prostheses. Such difference was statistically significant in zones 2, 3, and 4 (*P* < 0.05, see Table 3).

## 4. Discussion

Femoral neck fracture is a common type of fracture, and the pathogenic factors include osteoporosis, trauma, and femoral neck bone cyst. At present, there is a certain consensus on the application of HA in elderly patients with femoral neck fractures. The surgery can make patients achieve early ambulation, better solve the symptoms of hip joint pain and claudication caused by femoral head necrosis, avoid related complications caused by long-term bed rest, improve patients' QOL, and reduce family burden [5]. The controversy lies in the choice of the type of prosthesis in the surgical treatment of HA in elderly patients with femoral neck fractures. Periprosthetic stability is a key factor in the recovery and maintenance of postoperative hip function, and aseptic loosening of the bone-prosthesis interface is an important cause of postoperative pain, prosthesis loosening, and even periprosthetic fracture [6], and such loosening may be associated with periprosthetic bone loss. Bone loss after trauma and fracture surgery has been a research hotspot, and the rate of bone loss is influenced not only by friction-mediated osteolysis and stress shielding but also by the induction of osteoporosis with posttraumatic physiological alterations, thereby causing implant loosening [7]. Relevant studies have shown that the severity of trauma, intraoperatively excessive periosteal and vascular injury, and the level of functional recovery of the limb after surgery directly affect the extent of bone loss. In this study, 60 elderly patients with femoral neck fracture in our hospital were included as the study subjects, and all patients were treated with HA. The clinical application value of cemented and biologic prostheses was comprehensively evaluated by estimating the hip joint function and BMD values of two groups. By analyzing Table 1, it is found that there was no significant difference in clinical data such as age and BMI values between the two groups in addition to the surgery time (*P* > 0.05), which laid the foundation for the subsequent studies. In this study, trauma and surgical injuries were more similar between the two groups, but follow-up revealed some differences in patients' functional recovery outcomes, which might be associated with periprosthetic bone loss. The implanted biologic prosthesis was relatively unstable, possibly leading to postoperative pain, hindering patients' weight-bearing activity, and then affecting bone repair and resulting in low bone mass [8]. As Knutsen et al. reported [9], after total hip replacement surgery for patients with osteoarthritis of the hip using cemented and biologic prostheses, the degree of periprosthetic bone loss was different, which was obviously higher in biologic prostheses than in cemented prostheses, and the result was consistent with the study herein. In another study, quantitative computed tomography (QCT) measurements of BMD were performed after surgery in patients with femoral neck fractures of Garden types III and IV and hip osteoarthritis, and the Harris score was significantly lower, and bone loss was more obvious in the femoral neck fracture group compared with the hip osteoarthritis group. According to the study by Sinaki et al. [10], patients with femoral neck fracture more easily have bone mass loss after surgery, and such difference in bone mass loss is mainly caused by different functional recovery after surgery. Weight-bearing is an important driving force for bone formation, while dysfunction and non-weight-bearing are closely related to bone resorption. Good filling of the medullary cavity is an important factor for optimal weight-bearing stability of the prosthesis, whereas tight press-fit and solid initial fixations are essential requirements for achieving good long-term outcomes of the prosthesis, and long-term follow-up studies have found that patients achieve initial press-fit fixation by bone ingrowth or fibrous stable fixation in the proximal femur and isthmus [11]. The initial stability is very important, and the precise preparation of the femoral medullary cavity is an important factor to ensure the long-term stability of cementless prostheses. Elderly patients have osteoporosis with thinner femoral cortical and enlarged volume of the medullary cavity, it is not advisable to increase the template model blindly and sequentially during surgery, and C-arm X-ray fluoroscopy should be used to understand the filling rate of the prosthesis in the femoral medullary cavity when necessary. In addition, precise reaming helps to prevent the occurrence of anterior femoral fracture at the insertion of prosthesis and guarantees close contact between bone and prosthesis. Intraoperatively, when a suitable femoral template is punched to achieve a defined depth, C-arm X-ray fluoroscopy is performed to understand its position, depth, and fill rate in the medullary cavity, thus ensuring a good position and fill rate in the medullary cavity after prosthesis implantation. Cemented prostheses can be strongly bonded at the bone-prosthesis interface, offering a better biomechanical stabilization [12].

The cemented prosthesis can form the tiny noose with the surrounding residual cancellous bone and then firmly fix the prosthesis, which is helpful to maintain the initial stability of this prosthesis, with a relatively low risk of splitting in proximal femur during the implantation of prosthesis, and it can be completely filled with the marrow cavity to prevent the violent injury of the femoral stem prosthesis during the implantation. In addition, the biologic prosthesis can also carry out the coverage of bone cement according to the patients' anatomical structure of femur and leg length to make the prosthesis in the best position, but it cannot completely match the femur, so the effect of cemented prosthesis is better. In this study, Harris hip scores increased gradually in both groups at three follow-up visits after surgery, but the scores of the cemented prosthesis group were significantly higher than those of the biologic prosthesis group. Compared with the cemented prosthesis group, the reduction in BMD in the biologic prosthesis group was greater in all zones, with the most significant reductions in zones 2, 3, and 4. This difference may be because patients who underwent HA using cemented prostheses were able to achieve better functional recovery and weight-bearing exercises. Naturally, there are also disadvantages of using cemented prostheses for elderly patients, such as high risk of cardiopulmonary complications, long operation time, high technical requirements for surgery, and difficulties in secondary revision surgery [13]. And during the procedure, the use of cemented prosthesis implantation may increase the occurrence of periprosthetic fracture, air embolism, hypotension, and other complications [14]. Recently, studies have shown that with the advancement of medical devices as well as the improvement of surgical techniques, the incidence of the above complications has significantly decreased [15]. Compared with the use of biological prostheses in this study, the use of cemented prostheses increased surgery time by an average of 18 minutes, but the mean time of hospitalization and the incidence of postoperative related complications did not differ significantly between the two groups. Cemented prostheses have previously been documented to be preferred for the treatment of osteoporotic femoral neck fractures, but no further studies were performed [16]. Through the quantitative measurement of BMD, it is believed that cemented prostheses can provide early stabilization of the prosthesis-bone interface, which is good for functional recovery and reducing periprosthetic bone mass loss. Despite the constant progress of medical technology, there are still many difficulties in the treatment of femoral neck fractures, so that more efficient diagnosis and intervention modes need to be explored constantly by medical workers to provide more reliable basis for the treatment of femoral neck fractures. The contributions of this study were as follows. The application of the two types of prosthesis in HA was deeply understood by comparing the cemented and biologic prostheses, with a major progress and development in clinic and an extensive guiding significance in orthopedic treatment, which undoubtedly becomes the development direction of future medicine.

However, this study also has shortcomings that would affect the study results, such as the relatively small sample size, the differences in individual samples and prosthetic factors (the type of fracture, the preoperative osteoporosis condition, the impact of prosthetic model and geometry on stress shielding, etc.), and the differences in the surgical approach among the surgeons. Therefore, more prospective controlled studies are needed in the future.

## Figures and Tables

**Table 1:** Between-group comparison of patients' general data.

Group	*N* (cases)	Age (years)	BMI (kg/m^2^)	Surgery time (min)	Hospitalization time (d)
A	30	77.41 ± 5.65	22.64 ± 4.32	70.13 ± 3.50	8.82 ± 1.25
B	30	78.32 ± 4.73	21.37 ± 3.53	52.52 ± 3.60	8.52 ± 1.72
*t*	—	0.676	1.247	19.210	0.773
*P*	—	0.501	0.217	<0.001	0.443

**Table 2:** Between-group comparison of patients' postoperative Harris scores.

Postoperative Harris score	Group A	Group B	*t*	*p*
1 month	65.74 ± 5.32	63.43 ± 5.76	1.614	0.112
6 months	72.45 ± 6.53	67.23 ± 5.41	3.372	0.001
12 months	84.56 ± 6.70	80.29 ± 7.08	2.399	0.020

**Table 3:** Changes in mean BMD in the Gruen zones between the two groups at postoperative 1, 6, and 12 months.

Gruen zone	Group	1 month after surgery			6 months after surgery			12 months after surgery		
		(g/cm^2^)	*t*	*P*	(g/cm^2^)	*t*	*P*	(g/cm^2^)	*t*	*P*
R1	A	0.633 ± 0.110	0.407	0.685	0.595 ± 0.098	1.464	0.149	0.583 ± 0.001	1.962	0.071
	B	0.621 ± 0.118			0.557 ± 0.103			0.547 ± 0.021		
R2	A	1.417 ± 0.120	1.751	0.086	1.388 ± 0.125	2.649	0.01	1.305 ± 0.129	2.958	0.040
	B	1.351 ± 0.168			1.278 ± 0.190			1.194 ± 0.160		
R3	A	1.488 ± 0.126	2.284	0.026	1.456 ± 0.097	3.050	0.003	1.403 ± 0.114	3.679	0.001
	B	1.406 ± 0.151			1.347 ± 0.170			1.277 ± 0.149		
R4	A	1.615 ± 0.101	4.192	0.026	1.574 ± 0.106	3.765	<0.001	1.521 ± 0.136	4.146	<0.001
	B	1.489 ± 0.130			1.456 ± 0.135			1.377 ± 0.133		
R5	A	1.578 ± 0.128	0.335	0.738	1.555 ± 0.125	0.031	0.976	1.482 ± 0.136	0.339	0.736
	B	1.589 ± 0.126			1.556 ± 0.128			1.470 ± 0.138		
R6	A	1.364 ± 0.137	0.302	0.764	1.332 ± 0.130	0.406	0.686	1.275 ± 0.140	1.030	0.307
	B	1.375 ± 0.145			1.317 ± 0.155			1.234 ± 0.167		
R7	A	0.698 ± 0.134	1.550	0.127	0.676 ± 0.138	0.291	0.772	0.584 ± 0.112	1.245	0.218
	B	0.746 ± 0.104			0.666 ± 0.128			0.545 ± 0.130		

## Data Availability

Data to support the findings of this study is available on reasonable request from the corresponding author.
